# Nuclear export of VP19C is not essential for replication of herpes simplex virus type 1

**DOI:** 10.1186/2045-3701-4-55

**Published:** 2014-09-19

**Authors:** You Li, Dongwei Mao, Guoda Ma, Lili Cui, Hua Tao, Haihong Zhou, Wandong Liang, Bin Zhao, Keshen Li

**Affiliations:** Guangdong Key Laboratory of Age-Related Cardiac and Cerebral Diseases, Affiliated Hospital, of Guangdong Medical College, Zhanjiang, 524001 China; Gynaecological and obstetrical department of the fourth affiliated hospital of Harbin Medical University, Harbin, Heilongjiang 150001 China; State Key Laboratory of Virology, Wuhan Institute of Virology, Chinese Academy of Sciences, Wuhan, 430071 China; Renji College, Wenzhou Medical University, Wenzhou, 325000 China; Department of Neurology, Affiliated Hospital of Guangdong Medical College, Zhanjiang, 524001 China

**Keywords:** VP19C, Nuclear export signal (NES), Bacterial artificial chromosome (BAC), Replication

## Abstract

**Background:**

Herpes simplex virus (HSV) type 1 has a 152 kb double-stranded DNA genome that may encode more than 80 gene products, many of which remain uncharacterized. The HSV-1 triplex is a complex of three protein subunits, VP19C and a dimer of VP23 that is essential for capsid assembly. Previous studies have demonstrated that HSV-1 VP19C contains an atypical nuclear localization signal and a functional nuclear export signal (NES), which are both important for the nucleocytoplasmic shuttling of VP19C. However, whether the VP19C NES is required for efficient HSV-1 production is unknown.

**Findings:**

In the present study, a VP19C NES-mutated recombinant virus was generated by using bacterial artificial chromosome recombineering technology to investigate the role of VP19C nuclear export in HSV-1 replication. Our results demonstrate that the growth curves, plaque areas, subcellular localization and viral gene expression are indistinguishable between the VP19C NES-mutated virus and the wild-type virus.

**Conclusions:**

Our findings reported herein indicate abrogation of the nuclear export of VP19C did not affect HSV-1 replication and viral gene expression.

## Findings

### Construction of a VP19C NES-mutated BAC with recombineering technology

**Figure 1:**
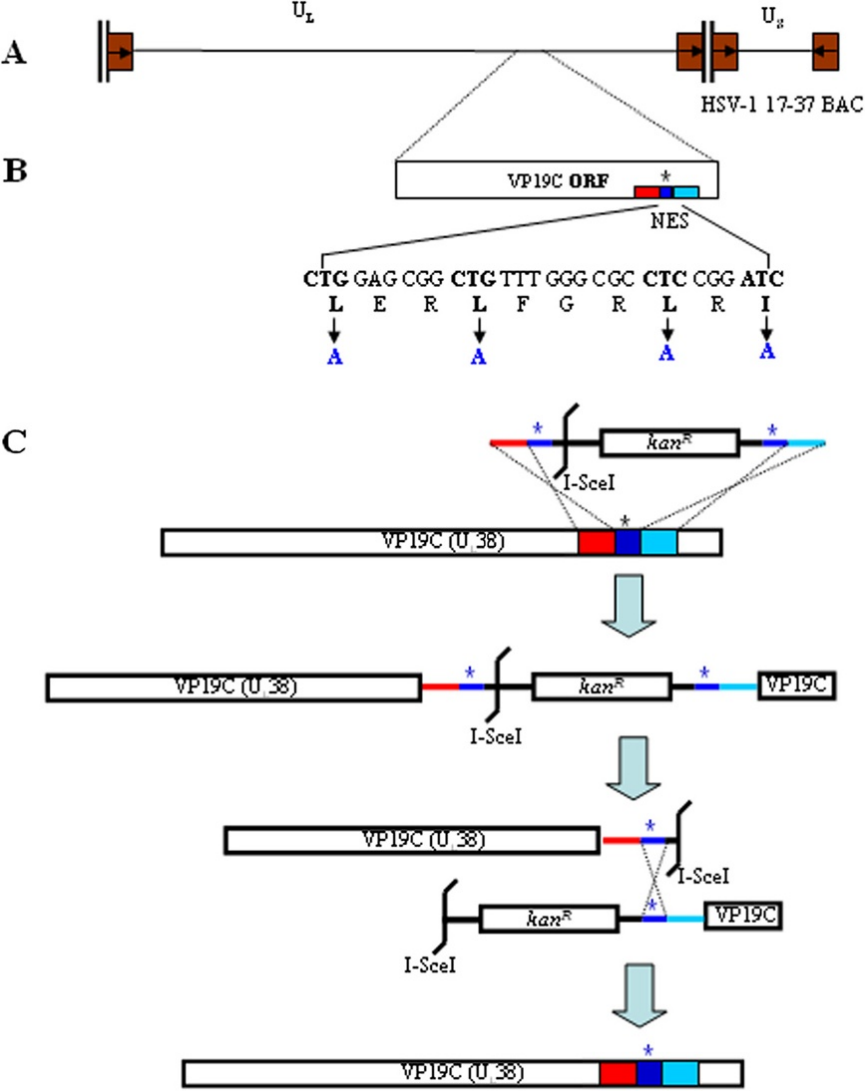
**Generation of VP19C NES mutants by mutagenesis. (A)** Schematic representation of the HSV-1 genome containing terminal long (TRL) and short (TRS) and internal long (IRL) and short (IRS) repeats flanking the unique long (U_L_) and short (U_S_) regions. **(B)** The NES mutations were introduced by using two-step red recombination. The sequences corresponding to the first (red and wathet) and second (blue) recombination events are shown in the indicated colors. **(C)** PCR products containing ~40 bp of homologous sequences upstream (red) and downstream (wathet) of the target loci, ~40 bp of complementary sequences (blue) encoding the mutations and I-SceI site, or the *Kan* ^*R*^ gene were electroporated into competent GS1783 *E. coli* containing the HSV-1 17-37BAC. After the first recombination, the PCR product was inserted into the homologous location within the *UL38* gene. After confirming that the PCR product was inserted into the correct locus by using RFLP and Southern blot hybridizations, the second recombination was performed in which the *Kan* ^*R*^ gene plus one of the complimentary homologous sequences were removed. The remaining genome contained the complete VP19C ORF with the corresponding mutations.

**Figure 2:**
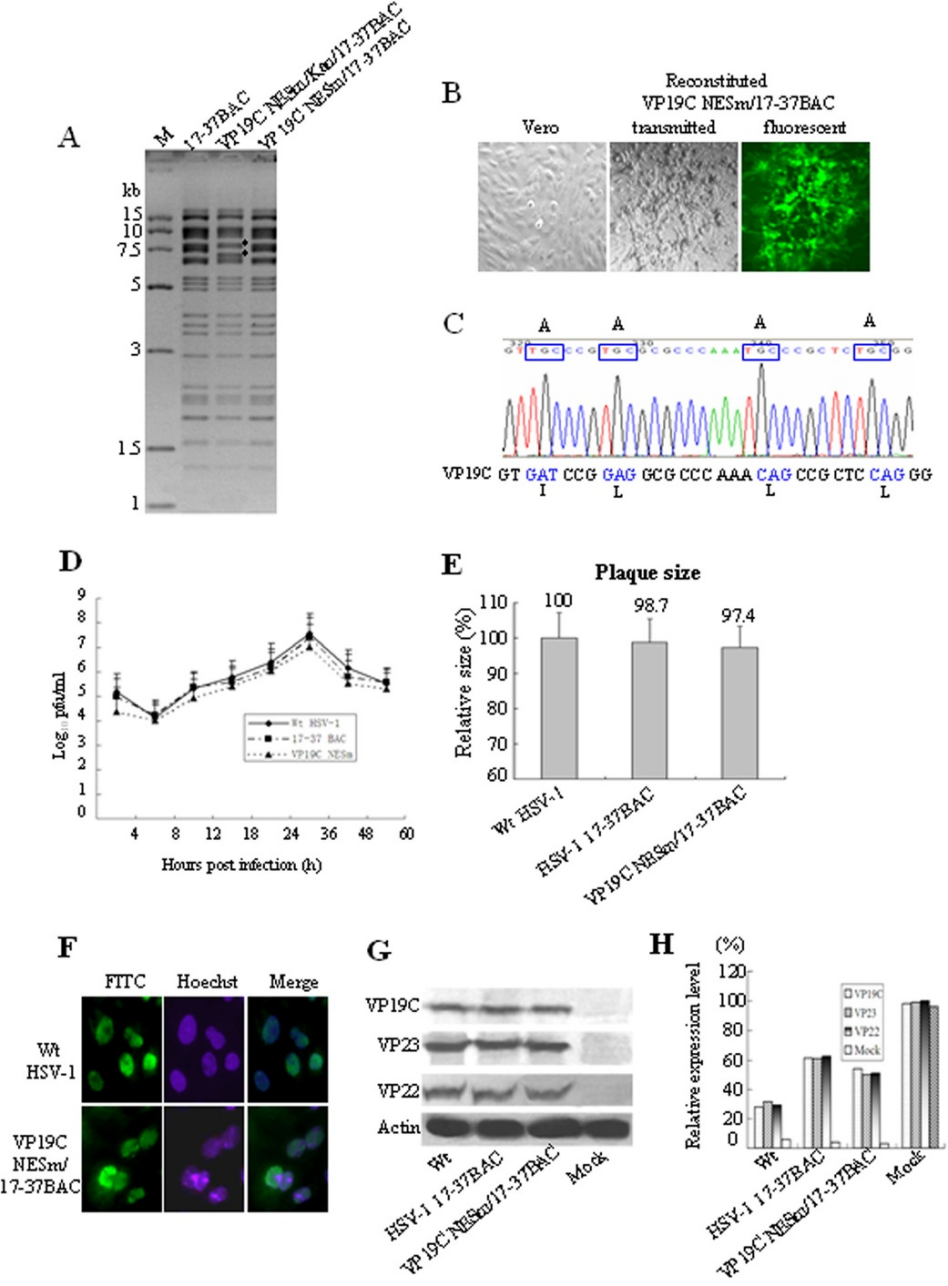
**Identification of the role of VP19C NES in HSV-1 life cycle. (A)** RFLP analysis of BAC recombinant variants. Parental p17-37BAC, pVP19C NESm/Kan/17-37BAC and pVP19C NESm/17-37BAC were digested with *Bam*HI. The sizes of the molecular markers are shown on the left. **(B)** VP19C NESm/17-37BAC recombinant virus reconstitution. pVP19C NESm/17-37BAC DNA electroporated into Vero cells caused cytopathic effects (CPE) (middle panel) with green fluorescence (right panel), while no CPE were observed in mock-transfected Vero cells (left panel). **(C)** The sequence of the VP19C NESm recombinant virus at the VP19C loci is shown. **(D)** The growth properties of wild-type HSV-1, parental 17-37BAC and VP19C NESm/17-37BAC were compared. Vero cells were infected with wild-type HSV-1, parental 17-37BAC or VP19C NESm/17-37BAC virus at an MOI of 1, and virus was harvested at the indicated time points and titrated on a Vero monolayer. The data plotted show the mean of three independent experiments. **(E)** Individual plaque area assays of wild-type, parental and recombinant VP19C NESm/17-37BAC virus are shown. The diameter of 80 plaques was measured for each virus, and the means ± standard deviations of the diameters were calculated and are shown. **(F)** Immunofluorescence assay for detecting the subcellular localization of VP19C in wild-type HSV-1 and VP19C NESm/17-37BAC virus-infected cells. Vero cells were infected with wild-type HSV-1 or VP19C NESm/17-37BAC virus at an MOI of 3 for 12 h and then probed with antibody against VP19C. **(G)** Western blot analysis of virion lysates was performed. Vero cells were infected with wild-type HSV-1, parental 17-37BAC or the VP19C NESm/17-37BAC virus at an MOI of 3. Expression was determined in cell lysates by Western blot analysis with antibodies against VP19C, VP22 and VP23. **(H)** The relative expression of virion proteins in the lysates was analyzed using Quantity One Imaging Software (Bio-Rad).

VP19C is a capsid protein of Herpes simplex virus (HSV) type 1, which is essential for the assembly of the capsid shell structure. If VP19C is absent, the capsid shells do not form. VP19C is reported to be essential for efficient transport of VP23 and VP5 to the nucleus, which is the site of capsid assembly [1]. A previous study revealed a leucine-rich sequcnce “^342^LERLFGRLRI^351^” in VP19C functioning as a nuclear export signal (NES) was responsible for its nucleocytoplasmic shuttling [2]. Replacement of key residues in the NES with alanines disrupts the shuttling of VP19C between the cytoplasm and nucleus. Cloning of the HSV-1 genome into infectious bacterial artificial chromosomes (BACs) has facilitated genetic manipulation of the HSV-1 genome in *Escherichia coli (E. coli)*. To investigate whether abrogation of VP19C nuclear export has an effect on HSV-1 lytic replication, a VP19C NES-mutated recombinant virus was constructed. Recombinant HSV-1 harboring the VP19C NES mutation was generated by a two-step Red-mediated recombination system in *E. coli* GS1783 strain using a HSV-1 17-37BAC [3]. The scheme of constructing a VP19C NES-mutated BAC was shown in Figure 1. Primer sequences used for BAC mutagenesis are listed in Table 1. Construction of the recombinant BAC pVP19C NESm/17-37BAC was performed as described previously [4]. The clones with the desired recombination were selected on kanamycin- and L-arabinose-containing plates and then subjected to PCR and restricted fragment length polymorphisms (RFLP) analysis. The mutant pVP19C NESm/17-37BAC showed BamHI digestion patterns similar to that of wild-type 17–37 BAC, while the pVP19C NESm/Kan/17-37BAC presented band variations approximately 6–8 kb fragments due to the insertion of a *Kan*^*R*^ cassette (Figure 2A). The restriction pattern and the BAC sequencing collectively indicate that no gross mutation was detected and the mutations occurred at the expected loci.

**Table 1:** **Primers used for generation of recombinant VP19C-NESm/17-37BAC**

Primers	Direction ^a^	Sequence ^b^
VP19C NESm	forward	CCGGCCGCAGAGCGGGCATTTGGGCGCGCACGGGCAACCAACACGATTCACGGCAC
	reverse	GTTGGTTGCCCGTGCGCGCCCAAATGCCCGCTCTGCGGCCGGCTCCAACCCG
VP19C NESm/BAC	forward	GCGCGGGTTGGAGCCGGCCGCGGAGCGGGCGTTTGGGCGCGCCCGGGCCACCAACACGATTCACGGCA***AGGATGACGACGATAAGTAGGG***
	reverse	CCGGGGGCGTCATGTCCTCGGTGCCGTGAATCGTGTTGGTGGCCCGGGCGCGCCCAAACGCCCGCTCCGC***CAACCAATTAACCAATTCTGATTAG***
BAC sequencing	reverse	TAACAACGGGGACGCTGACCG

^a^Directionality of the primer.

^b^Nucleotides in boldface type indicate priming sites within mutagenesis template plasmid pEP-KanS for amplification of *Kan* ^*R*^ cassette.

### Abrogation of VP19C nuclear export did not affect HSV-1 replication

To investigate the effect of the NES mutation of VP19C on the life cycle of HSV-1, 1–2 μg of recombinant pVP19C NESm/17-37BAC DNA was electroporated into Vero cells to reconstitute the VP19C NESm/17-37BAC virus (Figure 2B). Sequence analysis confirmed that the NES mutation in VP19C was not repaired during viral replication (Figure 2C), which indicated that the NES is not required for virus growth *in vitro*.

To compare the *in vitro* growth properties of VP19C NESm/17-37BAC virus with those of the parental virus and wild-type HSV-1, single step growth kinetics were determined as described previously [4]. As shown in Figure 2D, the VP19C NESm/17-37BAC virus showed growth kinetics almost identical to that of the parental 17-37BAC virus and wild-type HSV-1 on Vero cells (Figure 2D). Plaque diameters were measured following infection of Vero cells with VP19C NESm/17-37BAC, 17-37BAC or wild-type HSV-1 viruses in 6-well plates. There were no significant differences observed between the plaque diameters of the recombinant virus, the parental virus and the wild-type virus (Figure 2E).

### Abrogation of VP19C nuclear export did not affect subcellular localization of VP19C and viral gene expression

Immunofluorescence staining was applied to visualize the subcellular localization of VP19C in wild-type HSV-1 and VP19C NESm/17-37BAC virus-infected cells. As shown in Figure 2F, VP19C displayed a nuclear localization in VP19C NESm/17-37BAC virus-infected cells as it did in wild-type HSV-1-infected cells, indicating that the NES mutation of VP19C did not affect its nuclear localization in infection background.

Western blot was also performed to detect the expression of virion proteins VP19C, VP23 and VP22. As it is shown in Figure 2G and H, the expression of VP19C, VP23 and VP22 was unchanged. These results collectively indicated that abrogation of VP19C nuclear export does not affect subcellular localization of VP19C and viral gene expression.

## Discussion

The HSV-1 capsid is the most morphologically regular component of the virus structure. VP19C is an integral capsid protein that interacts with VP23 to form the triplex, which is the unit that forms the capsid. Capsid assembly of HSV-1 is known to take place in the nuclei of infected cells. It is reported that correct transport of the protein components to the site of capsid assembly is an important function of VP19C [5]. Because of its nuclear localization signal (NLS) and NES, VP19C is able to shuttle between the nucleus and the cytoplasm as part of a chromosome region maintenance 1 (CRM1) dependent pathway as well as through the atypical Ran- and importin β-dependent, but not importin α5, mechanisms [4]. These lines of evidence suggest that VP19C and VP23 may interact in the cytoplasm, and then, VP23 is translocated into the nucleus because of the NLS on VP19C to participate in capsid assembly.

The hydrophobic residues in the NES are important for nuclear export of VP19C, and VP19C is exported from the nucleus through the NES *via* a CRM1-dependent pathway. However, CRM1-mediated transport is highly subjected to various regulation processes, including the masking of NES, phosphorylation and heterodimerization of the protein and formation of disulfide bonds by an oxidative process [6]. Phosphorylation of residues in the vicinity of the NES and NLS may regulate the intracellular distribution of a protein [6]. For example, several viral proteins, such as the Rabies virus P-protein and chicken anemia virus VP3, regulate their NLS/NES activity *via* phosphorylation [6]. Additionally, the availability of specific cofactors may also influence this regulation. The mechanism by which subcellular localization is regulated and whether it is regulated over the course of HSV-1 infection will be interesting to determine.

BAC recombineering allows for the rapid and efficient modification of viral genomes in *E. coli*, thus providing an invaluable tool for the genetic manipulation of herpesvirus genomes and the production of altered forms of viruses to decipher the specific function of a gene in the virus life cycle and pathogenesis [7]. In the present study, we constructed a VP19C NES-mutated recombinant virus using BAC recombineering technology. The recombinant virus with the NES mutation in VP19C did not impair virus replication, and viral gene expression was also unaffected. Our previous study identified a non-classical NLS of VP19C, and the nuclear import of VP19C is required for efficient HSV-1 production [4]. These lines of evidence together suggest that the role of nuclear import and export may function differently in HSV-1 lytic replication. HSV-1 ICP27 was shown to shuttle between the nucleus and cytoplasm through a leucine-rich NES in a CRM1-dependent pathway [8]. However, ICP27 shuttling is not involved in viral DNA replication, but affects the expression of HSV-1 late genes [9]. Li *et al.* also identified a functional NLS and NES in ORF45 of Kaposi’s sarcoma-associated herpesvirus 8, and demonstrated that the NES is important for ORF45 nucleocytoplasmic shuttling. However, only the NLS-mutated virus had impaired virus production, and the NES-mutated virus did not affect viral replication [10]. Additionally, because VP19C could interact with other cellular proteins (such as nuclear pore complexs) or viral proteins (such as UL25) [4], it cannot be excluded that VP19C may export from the nucleus through other pathways in the context of infection. Although the NES seems dispensable for HSV-1 lytic replication in the present study, it might be important for certain ancillary functions that could facilitate its interaction with the host.

In conclusion, our study provides evidence that the nuclear export of VP19C is not essential for HSV-1 replication and viral gene expression. The potential role of nucleocytoplasmic shuttling of VP19C played in the HSV-1 viral life-cycle still needs to be further elucidated.

## Abbreviations

HSV-1: Herpes simplex virus type 1
NES: Nuclear export signal
NLS: Nuclear localization signal
BAC: Bacterial artificial chromosome
CRM1: Chromosome region maintenance 1
*E. coli*: *Escherichia coli*
RFLP: Restricted fragment length polymorphisms
Kan: Kanamycin.

## References

[1] Rixon FJ, Addison C, McGregor A, Macnab SJ, Nicholson P, Preston VG, Tatman JD (1996). Multiple interactions control the intracellular localization of the herpes simplex virus type 1 capsid proteins. J Gen Virol.

[2] Zhao L, Zheng C (2012). The first identified nucleocytoplasmic shuttling herpesviral capsid protein: herpes simplex virus type 1 VP19C. PLoS One.

[3] Gierasch WW, Zimmerman DL, Ward SL, Vanheyningen TK, Romine JD, Leib DA (2006). Construction and characterization of bacterial artificial chromosomes containing HSV-1 strains 17 and KOS. J Virol Methods.

[4] Li Y, Zhao L, Wang S, Xing J, Zheng C (2012). Identification of a novel NLS of herpes simplex virus type 1 (HSV-1) VP19C and its nuclear localization is required for efficient production of HSV-1. J Gen Virol.

[5] Adamson WE, McNab D, Preston VG, Rixon FJ (2006). Mutational analysis of the herpes simplex virus triplex protein VP19C. J Virol.

[6] Kutay U, Guttinger S (2005). Leucine-rich nuclear-export signals: born to be weak. Trends Cell Biol.

[7] Hall RN, Meers J, Fowler E, Mahony T (2012). Back to BAC: The Use of Infectious Clone Technologies for Viral Mutagenesis. Viruses.

[8] Lengyel J, Strain AK, Perkins KD, Rice SA (2006). ICP27-dependent resistance of herpes simplex virus type 1 to leptomycin B is associated with enhanced nuclear localization of ICP4 and ICP0. Virology.

[9] Rice SA, Lam V (1994). Amino acid substitution mutations in the herpes simplex virus ICP27 protein define an essential gene regulation function. J Virol.

[10] Li X, Zhu F (2009). Identification of the nuclear export and adjacent nuclear localization signals for ORF45 of Kaposi's sarcoma-associated herpesvirus. J Virol.

